# Schlemm’s Canal Endothelium Cellular Connectivity in Giant Vacuole and Pore Formation in Different Flow-type Areas: A Serial Block-Face Scanning Electron Microscopy Study

**DOI:** 10.3389/fcell.2022.867376

**Published:** 2022-04-13

**Authors:** David L. Swain, Senila Yasmin, Beatriz Fernandes, Ganimete Lamaj, Yanfeng Su, Haiyan Gong

**Affiliations:** ^1^ Department of Ophthalmology, Boston University School of Medicine, Boston, MA, United States; ^2^ Department of Anatomy and Neurobiology, Boston University School of Medicine, Boston, MA, United States; ^3^ The Affiliated Eye Hospital of Wenzhou Medical University, Wenzhou, China

**Keywords:** Schlemm’s canal endothelium, giant vacuoles, segmental aqueous humor outflow, serial block-face scanning electron microscopy, 3D electron microscopy, pores, cellular connectivity

## Abstract

Glaucoma is associated with increased resistance in the conventional aqueous humor (AH) outflow pathway of the eye. The majority of resistance is thought to reside in the juxtacanalicular connective tissue (JCT) region of the trabecular meshwork and is modulated by the inner wall (IW) endothelial cells of Schlemm’s canal (SC). The IW cells form connections with the underlying JCT cells/matrix, and these connections are thought to modulate outflow resistance. Two ways by which AH crosses the IW endothelium are through: 1) the formation of outpouchings in IW cells called giant vacuoles (GVs) and their intracellular pores (I-pores), and 2) intercellular pores between two adjacent IW cells (B-pores). AH outflow is segmental with areas of high-, low-, and non-flow around the circumference of the eye. To investigate whether changes in cellular connectivity play a role in segmental outflow regulation, we used global imaging, serial block-face scanning electron microscopy (SBF-SEM), and 3D reconstruction to examine individual IW cells from different flow areas of *ex vivo* perfused normal human donor eyes. Specifically, we investigated the differences in cellular dimensions, connections with JCT cells/matrix, GVs, and pores in SC IW cells between high-, low-, and non-flow areas. Our data showed that: 1) IW cell-JCT cell/matrix connectivity was significantly decreased in the cells in high-flow areas compared to those in low- and non-flow areas; 2) GVs in the cells of high-flow areas had significantly fewer connections beneath them compared to GVs in the cells of low- and non-flow areas; 3) Type IV GVs (with I-pores and basal openings) had significantly fewer connections beneath them compared to Type I GVs (no I-pore or basal opening). Our results suggest that a decreased number of cellular connections between the IW and JCT in high-flow areas is associated with increased numbers of GVs with I-pores and larger Type IV GVs observed in previous studies. Therefore, modulating the number of cellular connections may affect the amount of high-flow area around the eye and thereby modulate AH outflow.

## 1 Introduction

Primary open-angle glaucoma (POAG) is an optic neuropathy and a leading cause of irreversible blindness worldwide (Quigley, 1995). The only modifiable risk factor for POAG is intraocular pressure (IOP). Elevated IOP results from a disequilibrium between aqueous humor (AH) production and drainage (AGIS Investigators, 2000), which results from increased resistance to AH drainage in the trabecular outflow pathway. The majority of resistance is thought to reside in the juxtacanalicular connective tissue (JCT) region of the trabecular meshwork (TM) and is modulated by the inner wall (IW) endothelial cells of Schlemm’s canal (SC) (Grant, 1958, 1963; Maepea and Bill, 1989; Johnson et al., 1992; Maepea and Bill, 1992; Overby et al., 2009; Vahabikashi et al., 2019). The mechanisms by which the JCT and IW of SC interact and modify outflow resistance are still unclear.

The IW endothelium of SC is held together by tight junctions (Raviola and Raviola, 1981; Bhatt et al., 1995). One mechanism by which AH crosses the IW endothelium is through small openings, or intercellular pores, that form between adjacent IW cells (B-pores) (Ethier et al., 1998). Another mechanism by which AH passes through this endothelial monolayer is through giant vacuoles (GVs), which are outpouchings filled with AH that form by AH pushing against the basal side of the IW cells to form an invagination known as a basal opening (Tripathi, 1971). AH exits the GVs through intracellular pores (I-pores) on the luminal surface of the GV (Ethier et al., 1998). I-pores are thought to form in the cellular lining around the GVs as the GVs fill with AH and increase in size (Tripathi, 1972; Braakman et al., 2014). A previous study has shown that GVs with I-pores are larger and have thinner cellular lining around the GVs compared to GVs without I-pores (Swain et al., 2021). The I-pores allow AH to pass from the JCT through the GV and into the lumen of SC (Johnson and Erickson, 2000; Swain et al., 2021). The factors that influence GV and pore formation are not fully understood and warrant further investigation.

Previous studies have shown that flow of AH through the outflow pathway around the circumference of the eye is segmental, or non-uniform, by perfusing fluorescently-labeled tracers or dyes into enucleated eyes (Lu et al., 2008; Lu et al., 2011; Yang et al., 2013; Vranka et al., 2015; Cha et al., 2016; Huang et al., 2016; Ren et al., 2016; Swain et al., 2021) or living eyes (Huang et al., 2017a; Huang et al., 2017b; Huang et al., 2018). Previous studies of these specific areas of active and inactive flow have attempted to understand their morphological and molecular differences. One study examined TM endothelial cell specific differences using primary cells isolated from active and inactive areas and found differences in adhesion proteins and metalloproteinase inhibitors (Staverosky et al., 2020). Another study in our lab found that the number of GVs with I-pores and basal openings was greater in high-flow areas compared to low- and non-flow areas (Swain et al., 2021). However, these studies did not examine SC IW cells and their interactions with the underlying JCT cells and matrix.

Current treatment for POAG aims to maintain IOP within a normal range by decreasing AH production or increasing drainage of AH through uveal outflow pathways. A relatively new medication is netarsudil, a rho-kinase inhibitor, that targets the trabecular outflow pathway. Netarsudil has been shown to increase the amount of effective filtration area (EFA) around the circumference of the eye, which correlated with increased outflow facility (Ren et al., 2016). Other studies with rho-kinase inhibitors have shown that an increase in outflow facility is associated with physical separation between the IW of SC and the JCT in porcine, bovine, and monkey eyes (Rao et al., 2001; Lu et al., 2008; Lu et al., 2011), and expansion of the JCT in human eyes, which is also associated with increased active flow areas (Yang et al., 2013; Gong and Yang, 2014; Ren et al., 2016). One hypothesis is that the expansion observed with rho-kinase inhibitor treatment is due to the targeted release of cellular connections between SC IW cells and underlying JCT matrix and cells. Whether the connectivity between these cell types differs in active and inactive flow areas has not previously been investigated.

Previous transmission electron microscopy studies have categorized the various types of connections between SC IW endothelial cells and underlying JCT cells/matrix (Grierson et al., 1978; Lai et al., 2019). We previously used serial block-face scanning electron microscopy (SBF-SEM) to quantify the total number of connections on 3D-reconstructed individual IW cells and to investigate their differences between immersion- and perfusion-fixed (15 mmHg) eyes. We found that the number of IW-JCT connections significantly decreased in IW cells of perfusion-fixed eyes compared to IW cells from immersion-fixed eyes (Lai et al., 2019). Additionally, GVs were much larger and had I-pores in the IW cells from perfusion-fixed eyes, whereas no I-pores were found in IW cells from immersion-fixed eyes. However, this study did not compare differences between IW cells in different flow-type areas.

IW endothelial cells also connect to their adjacent IW cells through tight junctions (Raviola and Raviola, 1981; Bhatt et al., 1995). Studies have shown that the tight junctions simplify in response to increased pressure, and this simplification may promote B-pore formation and allow greater paracellular flow (Ye et al., 1997). The overlap between adjacent IW cells also decreases with increased pressure (Ye et al., 1997). Our previous study in immersion- and perfusion-fixed eyes found that B-pores only formed in areas where the overlap between adjacent IW cells was reduced to zero (Lai et al., 2019). However, this previous study did not examine the overlap between adjacent IW cells in different flow-type areas.

The aim of this study was to use SBF-SEM and subsequent 3D-reconstruction to investigate differences in cellular connectivity between 3D-reconstructed IW cells and their underlying JCT cells/matrix and between adjacent IW cells in high-, low-, and non-flow areas of human eyes perfusion-fixed at 15 mmHg, in order to understand how changes in cellular connections may play a role in GV and pore formation and the regulation of segmental outflow.

## 2 Materials and Methods

### 2.1 Human Donor Eyes

Two normal human donor eyes (Eye 1: 32 years-old; Eye 2: 80 years-old) without traceable identity and any known history of ocular disease or surgery were received from the Miracles in Sight Eye Bank (Winston-Salem, NC, United States) within 24 h post-mortem. All eyes were managed according to the Declaration of Helsinki guidelines on research involving human tissue and the Boston University Medical Center Institutional Review Board. The eyes were confirmed to be grossly normal under a dissecting microscope.

### 2.2 Ocular Perfusion and Fixation

The perfusion procedure has been described in detail previously (Scott et al., 2009). All eyes were perfused with Dulbecco’s phosphate-buffered saline (pH 7.3; Invitrogen, Grand Island, NY, United States) containing 5.5 mM d-glucose (collectively referred as GPBS) for 30 min at 15 mmHg using pressure-controlled hydraulic pumps to establish a stable baseline facility (Eye 1: 0.263 μL/min/mmHg; Eye 2: 0.318). Eyes were exchanged and perfused with a fixed volume (200 µL) of red fluorescent tracers (size: 200 nm; Catalog number: F8810; Thermo Fisher Scientific, Waltham, MA, United States) and perfusion-fixed at the same pressure with modified Karnovsky’s fixative (2% glutaraldehyde in 2% paraformaldehyde) for 30 min. Eyes were immersed in the same fixative overnight.

### 2.3 Global Imaging and Ocular Dissection

Global imaging was performed, as described previously (Cha et al., 2016) to visualize the outflow pattern along the entire TM and episcleral veins. Each fixed globe (*n* = 2) was cut into anterior and posterior segments through the equator, followed by careful removal of the lens, vitreous body, iris, cornea (with a 10-mm trephine), and excess conjunctiva. Anterior segments were imaged *en face* on both the TM and scleral surfaces with a fixed exposure time (5 s) using a 300 mm lens on a 4000 MP VersaDock imaging system (Bio-Rad Laboratories, Hercules, CA, United States) with Quantity One imaging software (Bio-Rad Laboratories).

From global images, flow-type areas were designated qualitatively based on the relative amount of fluorescence as follows: 1) *high-flow*, bright fluorescence on both anterior (episcleral veins) and posterior (TM) sides of anterior segments; 2) *low-flow*, intermediate fluorescence on both posterior and anterior sides; 3) *non-flow*, no visible fluorescence on either anterior or posterior sides. Global images of these eyes have been published previously (Swain et al., 2021). Two radial wedges (2 × 2 × 2 mm) of tissue from each flow-type area were shipped to Cleveland Clinic (Cleveland, OH, United States) for SBF-SEM.

### 2.4 Serial Block-Face Scanning Electron Microscopy and 3D Reconstruction

Wedges were processed for SBF-SEM using a previously described method (Deerinck et al., 2010; Swain et al., 2021). In brief, tissues were post-fixed with OsO4/K ferrocyanide and thiocarbohydrazide and *en-bloc* stained with uranyl acetate and lead aspartate. SC and the TM were identified in each block and imaged using a Zeiss Sigma VP serial block-face scanning electron microscope equipped with a Gatan 3View in-chamber ultramicrotome stage (Gatan, Inc., Pleasanton, CA, United States) and low-kV backscattered electron detectors optimized for 3View systems. Images were taken of each block face. Each image had a pixel size of 0.0101 µm × 0.0101 µm, and each section was 0.13 µm in thickness. The image field size was 141 μm x 60–71 µm. The total number of images was 11,334 for six blocks of tissue, and the mean number of images per sample was 1889. Image sets were compiled and aligned for analysis.

### 2.5 Morphometric Analyses

**FIGURE 1:**
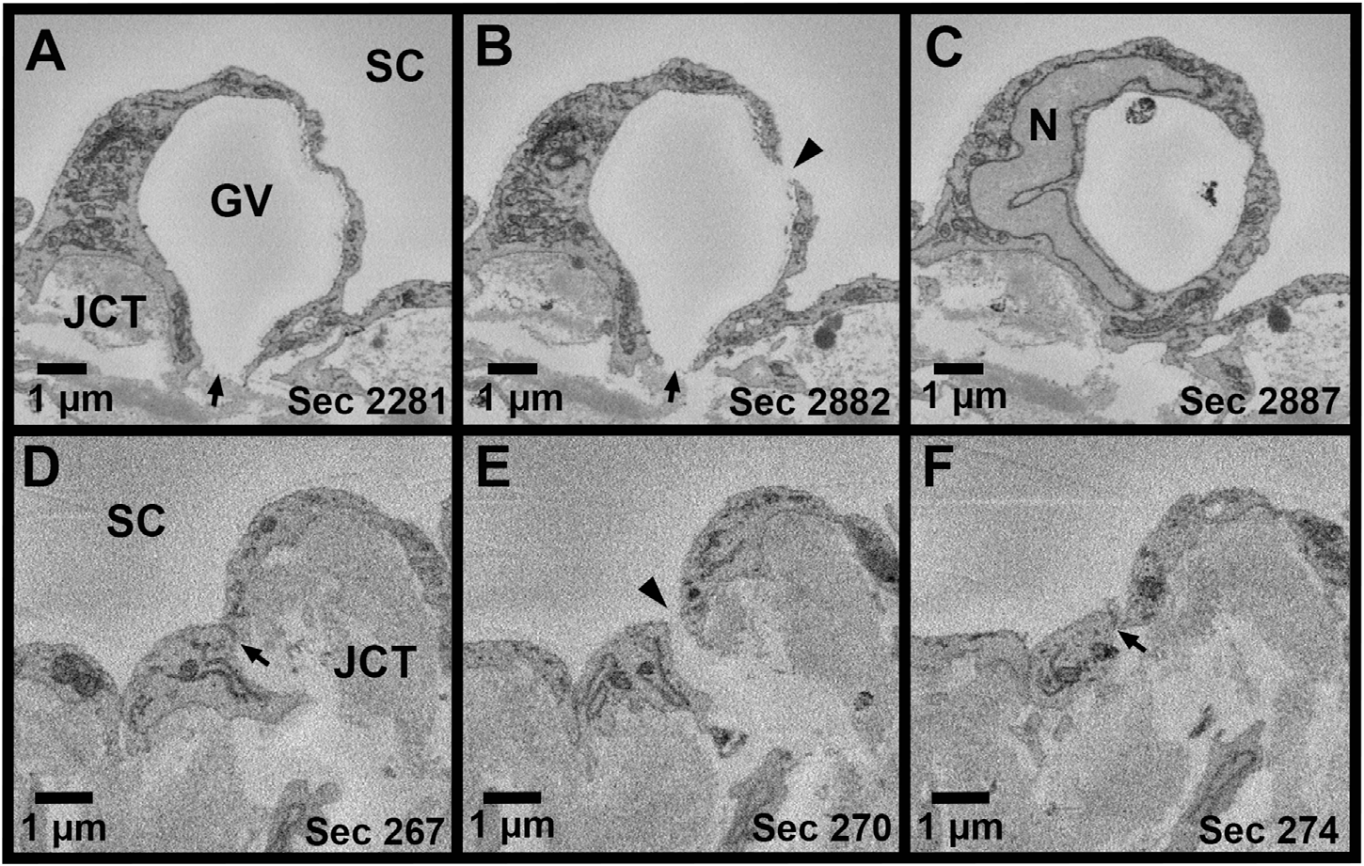
Giant vacuoles and two types of pores. **(A–C)**: Serial electron micrographs showing a giant vacuole (GV) with an I-pore (*arrowhead*) and basal opening (*arrow*). **(D–F)**: Serial electron micrographs showing a B-pore (*arrowhead*) between two adjacent inner wall cells. The cellular junction can be seen before and after the B-pore (*arrow*). SC = Schlemm’s canal; JCT = juxtacanalicular connective tissue; N = inner wall endothelial cell nucleus; Sec = serial block-face electron microscopy section number.

Using Reconstruct (Fiala, 2005), 11,334 SBF-SEM images were screened to identify SC IW cells (*n* = 15) from each flow-type area that were completely within the imaging field (total: 45 cells). All of the following morphometric analyses were performed twice, either by two investigators individually or by one investigator twice with 2 weeks between trials with less than 10% difference between trials or investigators (DLS, SY, BF, GL). For each IW cell, GVs were identified and counted (Figure 1A). I-pores were identified as openings in the cellular lining surrounding the GVs that had a clear separation with smooth borders (Figure 1B). Exclusion criteria included openings in the cellular lining that were in line with knife marks from the sectioning process or appeared rough with jagged edges. GVs were typed according to previous studies (Grierson and Lee, 1978; Koudouna et al., 2019; Swain et al., 2021): Type I: no basal opening or I-pore, Type II: basal opening, no I-pore; Type III: I-pore, no basal opening; Type IV: both basal opening and I-pore. The percentages of each GV type were determined. Volumes of GVs were determined from the manual segmentation using the Volume Tool in Reconstruct. B-pores were identified as clear openings between the reconstructed cell and either of its adjacent neighboring IW cells where the cell borders clearly separate (Figures 1D–F).

#### 2.5.1 Schlemm’s Canal Endothelial Cell Dimensions: Length, Width, Thickness, Volume

**FIGURE 2:**
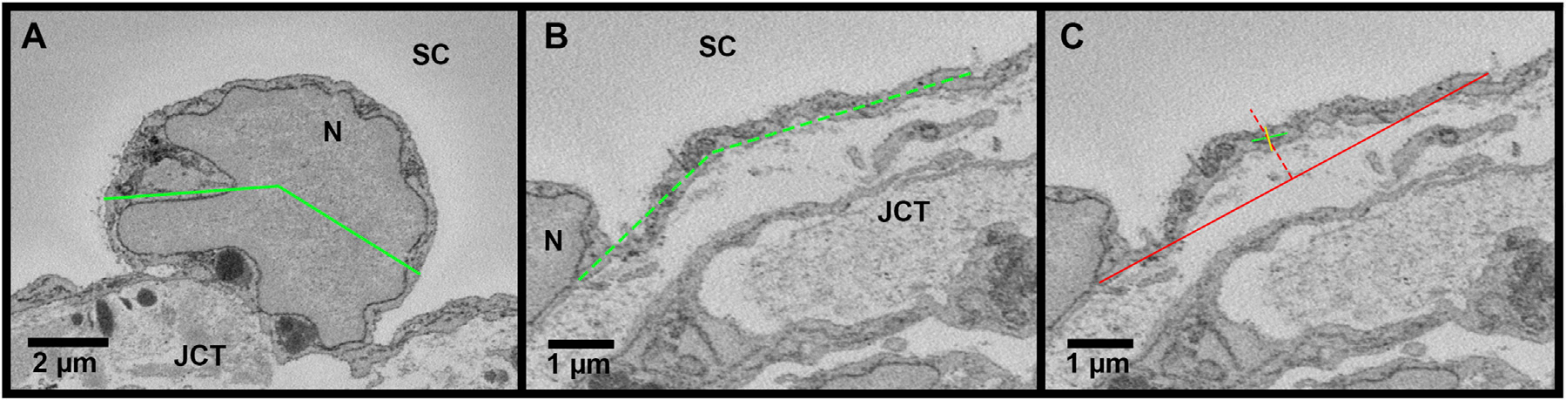
Methods for measuring cell width and thickness in Reconstruct. **(A)**: The cell width (*green line*) was measured in the nuclear area on the section with the greatest cross-sectional area of the nucleus (N). To account for the curvature of the inner wall, a segmented line was used, if necessary. **(B)**: The cell width was measured in the non-nuclear regions (*dashed green line*). **(C)**: The cell thickness was measured on the section with the greatest cross-sectional area of the nucleus to determine thickness in the nuclear region and on sections where the nucleus and giant vacuoles were not present (shown) to determine the thickness in non-nuclear thickness. First, a *solid red line* was drawn connecting the 2 cell borders and a *dotted red line* was drawn perpendicular at the halfway point. Then, a *solid green line* was drawn through the axis of the cell, intersecting the *dotted red line*. Finally, a *solid yellow line* was drawn perpendicular to the green axis line to measure the cell thickness, crossing the intersection of the *solid green* axis line and the *dotted red line*. JCT = juxtacanalicular connective tissue; SC = Schlemm’s canal.

Methods for measuring IW dimensions have been described previously (Lai et al., 2019). Using Reconstruct, cell length was measured for each reconstructed IW endothelial cell in the z-dimension, along the major axis, from the first image in which the cell appeared to the last section using the Z-trace function. The IW cell width was measured in two ways: 1) in *nuclear* regions, measured once on the section with the largest cross-sectional area (CSA) of the nucleus (Figure 2A); 2) in *non-nuclear* regions, measured every 40 sections throughout the length of the cell where the nucleus was not present (Figure 2B). To account for the curvature of the IW, a segmented line with up to three segments was used for IW cell width measurements. The IW cell thickness (i.e., basal-to-apical height) was measured in both nuclear and non-nuclear regions. In nuclear regions, one measurement was made on the section with the largest CSA of the nucleus. In non-nuclear regions, a measurement was made every 40 sections throughout the length of the cell where the nucleus and GVs were not present, and the average of these measurements was used to calculate the mean thickness per cell (Figure 2C). The IW cell volume was calculated using the Volume Tool in Reconstruct, and because the cell traces encompassed the GVs, the volumes of the GVs were subtracted to determine the cell volumes.

#### 2.5.2 IW/JCT Connections

**FIGURE 3:**
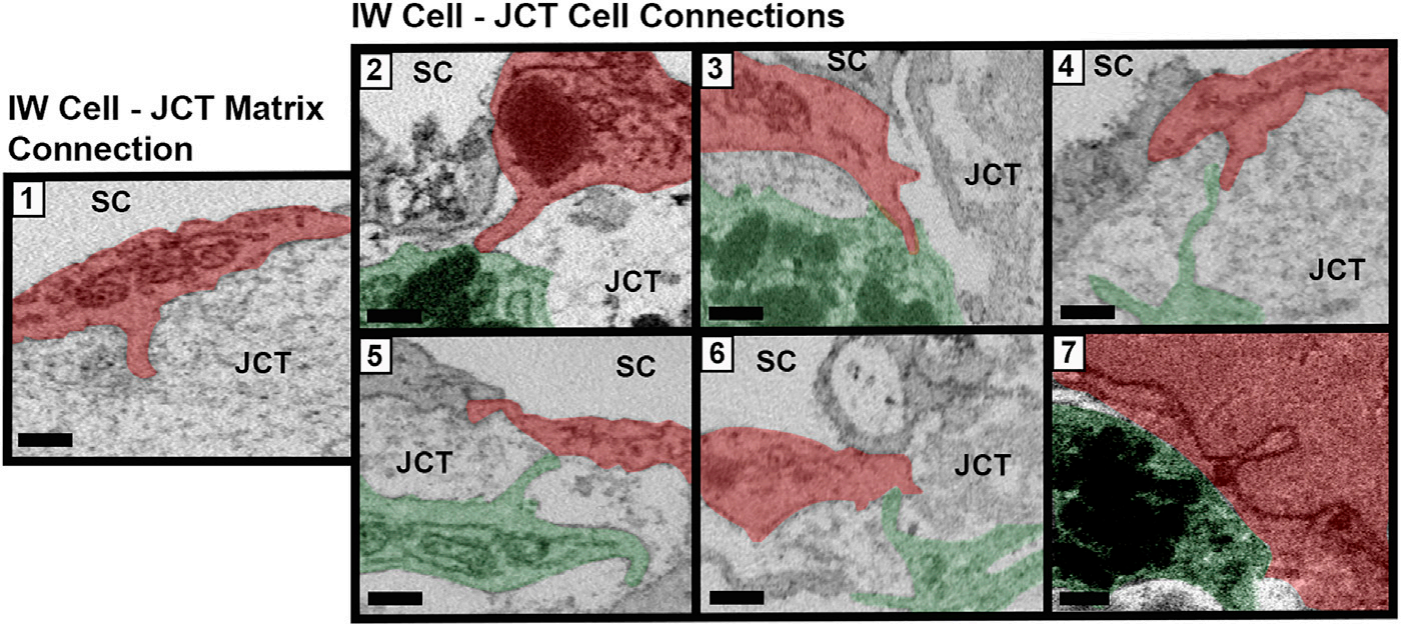
Types of cellular connections between IW endothelial cells and JCT cells/matrix. Types of connections between the inner wall (IW) endothelial cells (*red*) of Schlemm’s canal (SC) and underlying juxtacanalicular connective tissue (JCT) cells (*green*) and extracellular matrix. Type 1: IW cell process-to-JCT matrix; Type 2: IW cell process-to-JCT cell body; Type 3: IW tongue-in-JCT groove; Type 4: IW cell process-to-JCT cell process; Type 5: JCT process-to-IW-body; Type 6: JCT tongue-in-IW groove; and Type 7: IW body-to-JCT body. Scale bars = 0.5 µm.

Connections between SC IW cells and underlying JCT cells/matrix were manually counted for every cell. Connections were categorized into seven types, based on previous studies (Grierson et al., 1978; Lai et al., 2019): Type 1: IW cell process-to-JCT ECM; Type 2: IW cell process-to-JCT cell body; Type 3: IW cell tongue-in-JCT groove of cell body; Type 4: IW cell process-to-JCT cell process; Type 5: JCT cell process-to-IW cell body; Type 6: JCT cell tongue-in-IW groove of cell body; Type 7: IW cell body-to-JCT cell body (Figure 3). Types 1–6 were counted as connections if the projections from the cells had a height that measured at least 0.3 µm from the cell body, as measured on the SBF-SEM image in Reconstruct. Type 7 connections were counted if the area of contact was at least 0.5 µm^2^. For each GV for each cell, the number of total connections beneath the GVs was determined by examining each section in which the GV appeared and counting the connections. The ratio of the total number of connections beneath each GV to the GV volume was determined for GV types I-IV in order to examine the relationship between connections, GV size, and GV type (i.e., presence of an I-pore/basal opening). We accounted for volume, because previous studies have shown that these GV types have significantly different median volumes (Swain et al., 2021). Due to the relatively small number of GV Types III and IV in different flow-type areas, this analysis was conducted using all GVs from all cells.

#### 2.5.3 IW/IW Overlap and B-Pores

**FIGURE 4:**
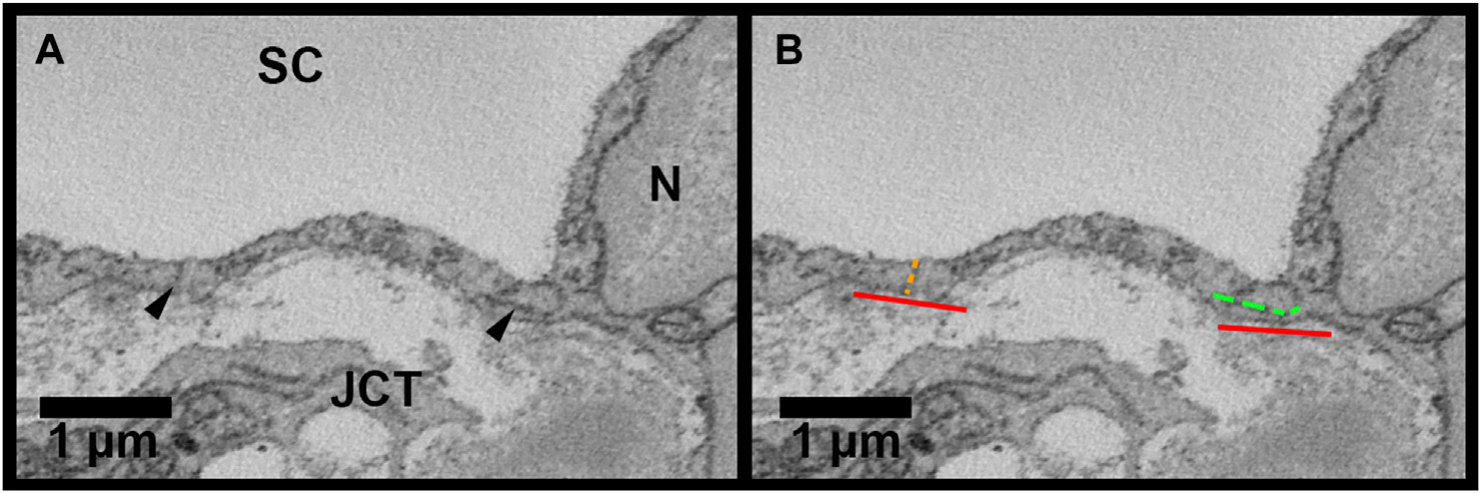
Methods for inner wall cell overlap length measurements in Reconstruct. **(A)**: Inner wall cell overlap length (OL) was measured on both sides of the reconstructed cells on every 40 sections throughout the length of each cell. The junctions between the cell and its adjacent cells are demarcated with *arrowheads*. **(B)**: To measure OL, a line parallel to the inner wall (IW) was drawn first (*red lines*). OL was measured if the plane of overlap was parallel to the axis of the inner wall or within 45° of the IW axis (example shown by the *segmented green line* on the right of the cell). An example of no overlap length (OL = 0 µm) is shown on the left of the cell (*segmented orange line*). JCT = juxtacanalicular connective tissue; SC = Schlemm’s canal; N = inner wall endothelial cell nucleus.

The connectivity between adjacent IW cells was quantified by measuring the overlap length (OL). Overlap length between the reconstructed IW cells and their adjacent IW cells was measured for each cell on every 40 sections through the entire cell length on both sides of the cell. These values were averaged to find the mean OL for each cell. On each image, OL was measured if the borders of the adjacent IW cell were parallel or within 45° of the axis of the IW (Figure 4). Cell borders that were perpendicular or at least >45° away from the axis of the IW were not counted as OL. For each B-pore, OL was also measured on sections before and after the pore was present to determine the amount of OL around B-pores. This OL was compared to the average OL for each cell on which the B-pore was found.

### 2.6 Statistical Methods

All data are listed as mean ± SEM, except GV volumes, which are reported as medians and interquartile ranges (IQR). All statistical analyses were performed using R statistical computing package (v3.5.1; R Foundation for Statistical Computing, Vienna, Austria). One-way ANOVA tests were performed to analyze differences in cell dimensions, connections, and overlap between flow-type areas with post-hoc Tukey HSD tests for pairwise comparisons. For ratios of total cellular connections beneath a GV-to-GV volume, Kruskal-Wallis tests were used with post-hoc Wilcoxon rank-sum tests for pairwise comparisons between GV types, due to small sample size for type III GVs.

## 3 Results

**FIGURE 5:**
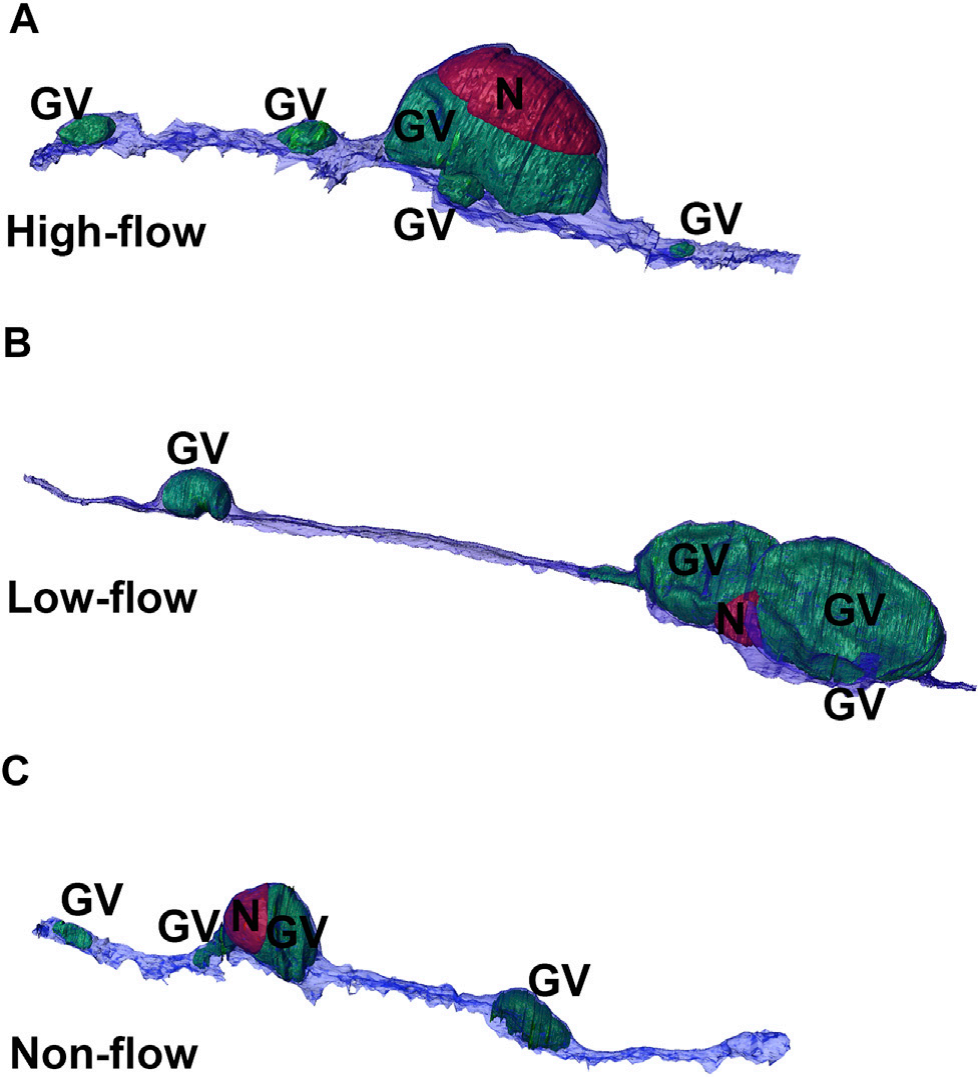
3D reconstructions of cells from three flow-type areas. Representative 3D reconstructions of Schlemm’s canal inner wall endothelial cells from high-**(A)**, low-**(B)**, and non-flow **(C)** areas. Cells typically had one or two large giant vacuoles (GVs, *green*) near the nucleus (N, *red*) with smaller GVs throughout the length of the cell.

Overall, a total of 45 IW cells were reconstructed (15 from each flow-type area). These cells had 234 GVs, 41 I-pores, and 12 B-pores with adjacent IW cells. These counts, mean pores/cell, and percentages of GVs with I-pores in three different flow-type areas are summarized in Table 1. Examples of 3D-reconstructed cells are shown in Figure 5.

**TABLE 1:** Summary of giant vacuoles and pores of reconstructed IW cells.

Flow-Type	Count: IW Cells	Count: GVs	Mean #GVs/Cell (SEM)	Count: I-Pores (Mean/Cell)	Count: GVs with I-Pores (% of Total)	Count: B-Pores	Count: IW cells with a B-Pore
High-flow	15	105	7.0 (1.2)	20 (1.3)	20 (19.0%)	3	3
Low-flow	15	66	4.4 (0.6)	15 (1.0)	14 (21.2%)	2	2
Non-flow	15	63	4.2 (0.6)	6 (0.4)	6 (9.5%)	7	4
Overall	45	234	5.2 (0.5)	41 (0.9)	40 (17.1%)	12	9

### 3.1 IW Cell Dimensions: Length, Width, Thickness, and Volume

#### 3.1.1 Length

**FIGURE 6:**
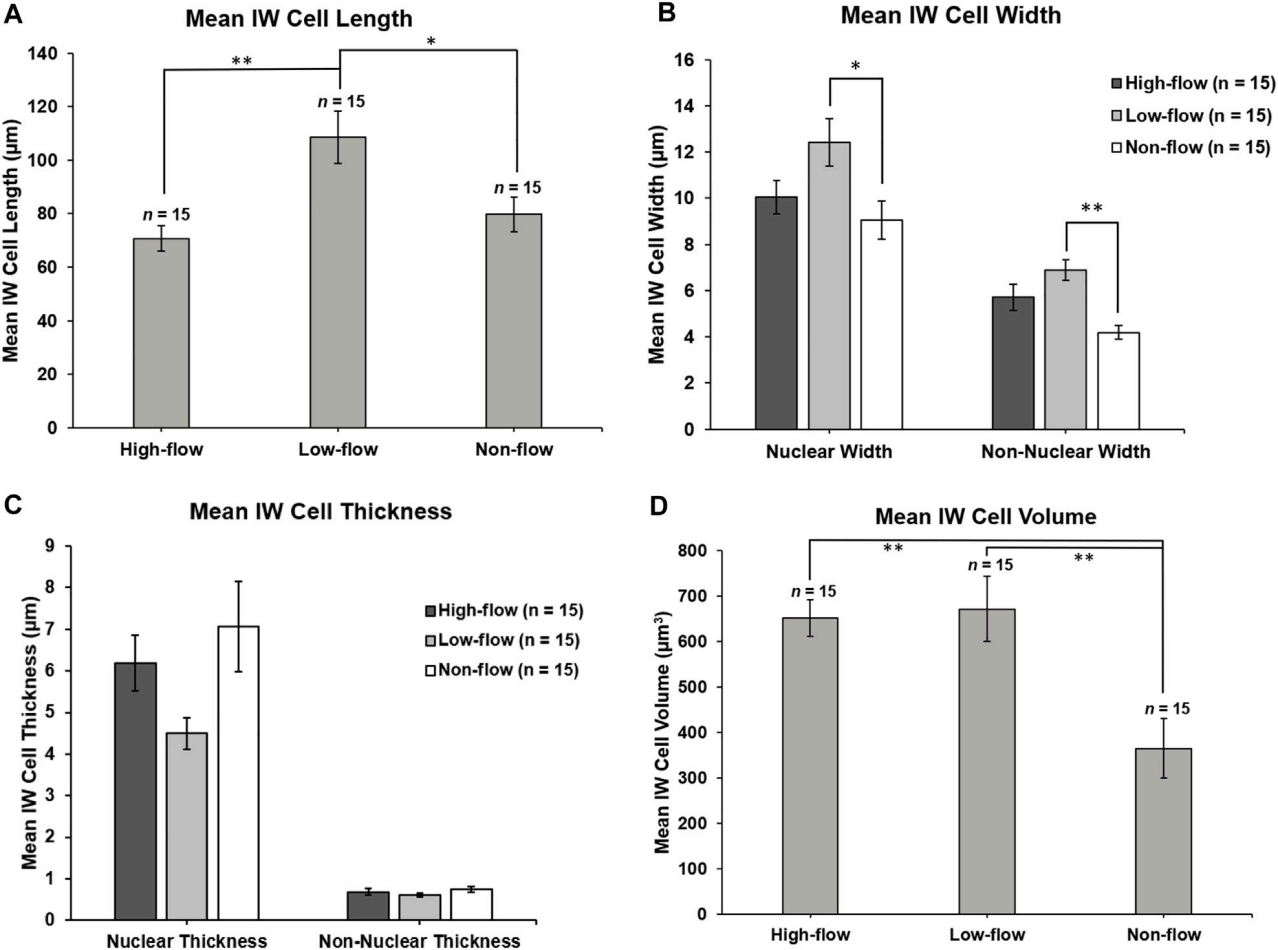
Inner wall cell dimensions. **(A)**: Mean IW cell length was measured in the z-plane through the stack of serial images. IW cells in low-flow areas were significantly longer than IW cells in high- (*p* = 0.02) and non-flow areas (*p* ≤ 0.01). **(B)**: Mean nuclear IW cell width was measured on the section with the largest cross-sectional area of the nucleus. In nuclear regions, IW cells were significantly wider in low-flow areas than in non-flow areas (*p* = 0.02). Non-nuclear IW cell width was measured on sections where the nucleus was not present. In non-nuclear regions, IW cells were significantly wider in low-flow areas than non-flow areas (*p* ≤ 0.01). **(C)**: Mean IW cell thickness (basal-to-apical height) in nuclear regions was not significantly different among cells in different flow-type areas (*p* = 0.06). Mean IW cell thickness in non-nuclear regions was not significantly different among cells in different flow-type areas (*p* = 0.24). **(D)**: Mean IW cell volume was calculated by subtracting the volume of giant vacuoles from the cell traces. Mean volumes of IW cells in high- and low-flow areas were significantly larger than IW cells in non-flow areas (both *p* ≤ 0.01). *p < 0.05; **p ≤ 0.01. *Error bars*: SEM.

Mean length of SC IW cells was 70.76 ± 4.78 µm, 108.56 ± 9.68 µm, and 79.68 ± 6.38 µm in high-, low-, and non-flow areas, respectively (Figure 6A). Mean lengths differed significantly by flow-type area (ANOVA, *p* ≤ 0.01). Specifically, the cells in the low-flow area were significantly longer than those in high- (*p* = 0.02) and non-flow areas (*p* ≤ 0.01).

#### 3.1.2 Width

The mean width of SC IW cells measured at the section with the largest cross-sectional area of the nucleus (nuclear width) was 10.05 ± 0.73 µm, 12.41 ± 1.03 µm, 9.06 ± 0.82 µm in high-, low-, and non-flow areas, respectively, and these differed significantly (ANOVA, *p* = 0.03; Figure 6B). Specifically, in nuclear regions, the IW cells were significantly wider in low-flow areas than those in non-flow areas (*p* = 0.02). The mean non-nuclear width of SC IW cells was 5.72 ± 0.56 µm, 6.90 ± 0.45 µm, 4.18 ± 0.30 µm in high-, low-, and non-flow areas, respectively, and these differed significantly (ANOVA, *p* ≤ 0.01; Figure 6B). Specifically*,* in non-nuclear regions, IW cells in low-flow areas were significantly wider than the cells in non-flow areas (*p* ≤ 0.01). The mean non-nuclear width of IW cells in high-flow areas was not quite significantly wider compared to IW cells in non-flow areas (*p* = 0.05).

#### 3.1.3 Thickness

The mean thickness of SC IW cells in nuclear regions was 6.19 ± 0.67 µm, 4.50 ± 0.38 µm, 7.07 ± 1.08 µm in high-, low-, and non-flow areas, respectively, and these did not differ significantly (ANOVA, *p* = 0.06; Figure 6C). The thickness of SC IW cells in non-nuclear regions was 0.68 ± 0.08 µm, 0.62 ± 0.04 µm, 0.75 ± 0.07 µm in high-, low-, and non-flow areas, respectively, and these did not differ significantly (ANOVA, *p* = 0.24; Figure 6C).

#### 3.1.4 Volume

Mean volumes of SC IW cells were 669.58 ± 65.50 µm^3^, 671.96 ± 65.06 µm^3^, 383.25 ± 51.53 µm^3^ in high-, low-, and non-flow areas, respectively. Mean volumes varied significantly among flow-type areas (ANOVA, *p* ≤ 0.01). Specifically, the cells in high- and low-flow areas had significantly larger cellular volumes than the cells in non-flow areas (both *p* ≤ 0.01; Figure 6D).

### 3.2 IW/JCT Connections

#### 3.2.1 Total Cellular Connections Between SC IW Cells and JCT Cells/Matrix

**FIGURE 7:**
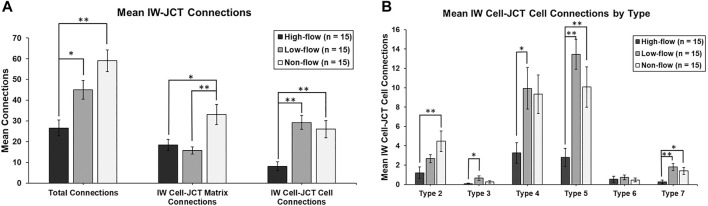
Mean number of cellular connections between IW cells and JCT cells/matrix. **(A)**: Total connections were significantly decreased in cells in high-flow areas, compared to those in low- and non-flow areas. When analyzed by IW cell to JCT matrix (cell-matrix connections), IW cells in high-flow and low-flow areas had significantly fewer connections than those in non-flow areas. When analyzed by IW cell to JCT cells (cell-cell connections), IW cells in high-flow areas had significantly fewer connections than those in low- and non-flow areas. **(B)**: When IW cell-JCT cell connections were analyzed by type, the most common types were type 5, 4, and then 2. IW cells in high-flow areas had significantly fewer connections than cells in low-flow, non-flow, or both for all types, except type 6. Specifically, for type 2, IW cells in high-flow areas had significantly fewer connections than IW cells in non-flow areas. For type 3, IW cells in high-flow areas had significantly fewer connections than IW cells in low-flow areas. For type 4, IW cells in high-flow areas had significantly fewer connections than IW cells in low-flow areas. For type 5, IW cells in high-flow areas had significantly fewer connections than IW cells in low- and non-flow areas. For type 7, IW cells in high-flow areas had significantly fewer connections compared to IW cells in low- and non-flow areas. *p < 0.05; **p ≤ 0.01. *Error bars*: SEM.

The mean total number of connections per IW cell between IW cells and JCT cells/matrix were 26.6 ± 3.8, 45.0 ± 4.5, and 59.1 ± 5.2 in high-, low-, and non-flow areas, respectively. These varied significantly among flow-type areas (ANOVA, *p* ≤ 0.01). Specifically, the cells in high-flow areas had significantly fewer total connections than the cells in low- (*p* = 0.02) and non-flow areas (*p* ≤ 0.01; Figure 7A). No significant difference in the mean total connections was found between the cells in low- and non-flow areas (*p* = 0.08; Figure 7A).

##### 3.2.1.1 IW Cell-JCT Matrix Connections

Mean IW cell-JCT matrix connections per IW cell were 18.5 ± 2.7, 15.7 ± 1.7, and 33.1 ± 4.9 in high-, low-, and non-flow areas, respectively (Figure 7A). These varied significantly among flow-type areas (ANOVA, *p* ≤ 0.01). Specifically, the cells in high- and low-flow areas had significantly fewer IW cell-JCT matrix connections compared to the cells in non-flow areas (*p* = 0.01 and *p* ≤ 0.01, respectively; Figure 7A).

##### 3.2.1.2 IW Cell-JCT Cell Connections

Mean IW cell-JCT cell connections per IW cell were 8.1 ± 2.2, 29.3 ± 3.4, and 26.0 ± 4.1 in high-, low-, and non-flow areas, respectively (Figure 7A). These varied significantly among flow-type areas (ANOVA, *p* ≤ 0.01). Specifically, the cells in high-flow areas had significantly fewer IW cell-JCT cell connections compared to those in low- and non-flow areas (both *p* ≤ 0.01; Figure 7A).

IW cell-JCT cell connections were analyzed by their types (2–7) based on how they connect to one another. The most common types of IW cell-JCT cell connections were types 5, 4, and then 2. Mean number of IW-cell-JCT cell connections per IW cell differed significantly among three flow-types for type 2 (ANOVA, *p* = 0.01), type 3 (*p* = 0.03), type 4 (*p* = 0.02), type 5 (*p* ≤ 0.01), and type 7 (*p* ≤ 0.02) (Figure 7B). Only type 6 connections did not differ significantly among three flow-type areas (*p* = 0.74). Generally, the cells in high-flow areas had significantly fewer connections than those in low-flow, or non-flow, or both. Specifically, for type 2, the cells in high-flow areas had significantly fewer connections (1.2 ± 0.6) than the cells in non-flow areas (4.5 ± 1.1; *p* ≤ 0.01). For type 3, the cells in high-flow areas had significantly fewer connections (0.1 ± 0.1) than those in low-flow areas (0.7 ± 0.2; *p* = 0.02). For type 4, the cells in high-flow areas had significantly fewer connections (3.3 ± 1.1) than those in low-flow areas (9.9 ± 2.2; *p* = 0.03). For type 5, the cells in high-flow areas had significantly fewer connections (2.8 ± 0.9) than those in low-flow (13.5 ± 1.6; *p* ≤ 0.01) and non-flow areas (10.1 ± 2.1; *p* ≤ 0.01). For type 7, the cells in high-flow areas had significantly fewer connections (0.3 ± 0.2) compared to those in low-flow (1.8 ± 0.4; *p* ≤ 0.01) and non-flow areas (1.4 ± 0.4; *p* = 0.03) (Figure 7B).

#### 3.2.2 Giant Vacuoles and IW Cell-JCT Cell Connections Beneath GVs

The mean numbers of GVs per IW cell were 7.0 ± 1.2, 4.4 ± 0.6, and 4.2 ± 0.6 in high-, low-, and non-flow areas, respectively (Table 1). These means were not quite significantly different (*p* = 0.05); however, the range of number of GVs per IW cell was highest in high-flow (range: 1–15), then low-flow (range: 1–11), and then smallest in non-flow (range: 1–9). The percentages of GVs with I-pores appeared larger in the cells in high- (19.0%) and low-flow (21.2%) areas compared to non-flow (9.5%) (Table 1).

**FIGURE 8:**
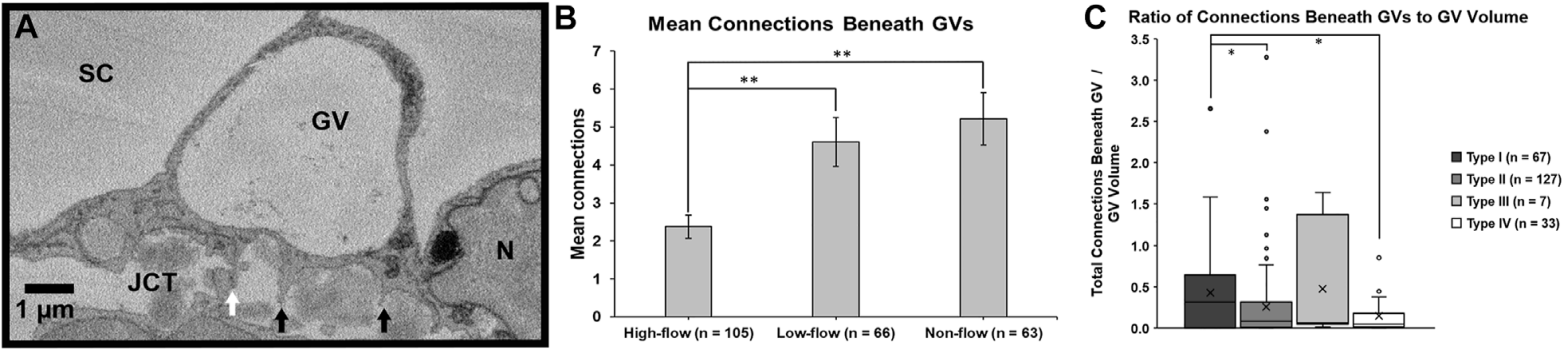
Mean connections beneath giant vacuoles. **(A)**: Connections between Schlemm’s canal (SC) endothelial cells and underlying juxtacanalicular connective tissue (JCT) cells (*white arrow*) and matrix (*black arrows*) were identified beneath each giant vacuole (GV) and counted. N = endothelial cell nucleus. **(B)**: The mean number of connections beneath GVs in the cells in high-flow areas was significantly fewer compared to GVs in the cells in low- or non-flow areas (both *p* ≤ 0.01). *Error bars*: SEM. **(C)**: When standardized by volume and analyzed by type, Types IV and II GVs had significantly fewer connections per unit volume compared to Type I GVs (both *p* = 0.02). Type I: GV with no basal opening or I-pore; Type II: GV with a basal opening, no I-pore; Type III: GV with an I-pore, no basal opening; Type IV: GV with both basal opening and I-pore. *Whiskers* = 1.5 interquartile range (IQR). *X* = mean. *p < 0.05; **p ≤ 0.01.

For each GV of each IW cell, we determined the number of connections that were beneath the GVs in each SBF-SEM image in which the GVs appeared. We found that the mean number of connections beneath a GV differed significantly among flow-type areas (*p <* 0.01). Specifically, the GVs in high-flow areas had significantly fewer connections beneath them (2.4 ± 0.3; *n* = 105) compared to GVs in low-flow (4.6 ± 0.6; *n* = 66; *p* ≤ 0.01) and non-flow areas (5.2 ± 0.7; *n* = 63; *p* ≤ 0.01; Figures 8A, B). We also calculated the percentage of connections under GVs for each IW cell. We found that a mean of 39.1 ± 2.7% of connections was under GVs for our 45 cells, compared to 60.9% of connections in areas without GVs. This did not differ among flow-type areas (44.6 ± 5.4% in high-flow, 41.2 ± 5.4% in low-flow, and 31.6 ± 3.7% in non-flow areas) (*p* = 0.12).

We also determined the percentages of each GV type, based on previous studies (Grierson and Lee, 1978; Koudouna et al., 2019; Swain et al., 2021): Type I: no basal opening or I-pore; Type II: basal opening, no I-pore; Type III: I-pore, no basal opening; and Type IV: both basal opening and I-pore. Of the 234 GVs we found in all 45 cells, 67 (28.6%), 127 (54.3%), 7 (3.0%), 33 (14.1%) were Types I, II, III, and IV GVs, respectively. Due to the relatively small numbers of types III and IV across different flow areas, the subsequent analysis was conducted using all GVs combined.

To investigate the relationship between cellular connections and GV types, we determined the median ratio of total connections beneath GVs to GV volume for the four types of GVs. The medians were 0.32 (IQR: 0.01–0.63), 0.08 (IQR: 0.01–0.31), 0.06 (IQR: 0.05–0.75), and 0.04 (IQR: 0.01–0.14) in GV types I, II, III, and IV, respectively. We found that these ratios varied significantly among GV types (Kruskal-Wallis test, *H* = 7.84, *p* < 0.05). Specifically, the median ratios of total connections per unit GV volume in Type IV (with I-pore and basal opening) and Type II (basal opening, no I-pore) GVs were significantly decreased compared to Type I (no basal opening or I-pore) (both *p* = 0.02; Figure 8C).

### 3.3 IW/IW Overlap and B-Pores

**FIGURE 9:**
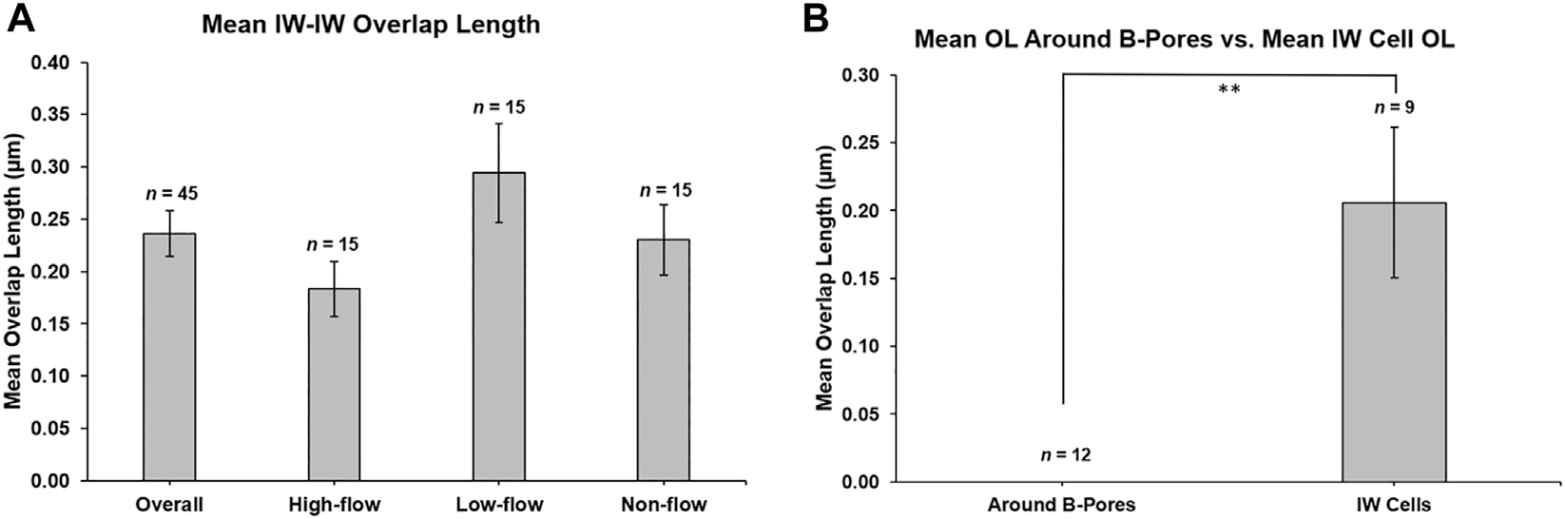
Mean overlap length between adjacent inner wall cells. **(A)**: Mean overlap length (OL) was 0.236 ± 0.022 µm (*n* = 45 cells), and this was not different between flow-type areas. **(B):** OL was measured on the sections before and after a B-pore was observed for 12 B-pores. OL was always 0 μm, which was significantly less (*p* ≤ 0.01) than the mean OL for the nine inner wall (IW) cells on which the B-pores were found. **p ≤ 0.01. *Error bars*: SEM.

Mean OL was 0.24 ± 0.02 µm for all 45 reconstructed cells. Mean OL was 0.18 ± 0.03 µm, 0.29 ± 0.05 µm, and 0.23 ± 0.03 µm for high-, low-, and non-flow cells, respectively (Figure 9A). OL did not differ significantly between flow-type areas (*p* = 0.12). There were 12 B-pores in total, three in high-flow, two in low-flow, and seven in non-flow. Nine of our 45 IW cells had at least one B-pore. In non-flow, 1 cell had 2 B-pores and 1 cell had 3 B-pores with adjacent cells. OL near B-pores (measured on sections before and after a B-pore was present) was always 0 µm for all 12 B-pores, and this was significantly less than the mean OL for these nine cells (0.21 ± 0.06 µm, *p* ≤ 0.01; Figure 9B).

## 4 Discussion

In this study, we used SBF-SEM and 3D reconstruction to investigate the cellular connectivity between IW endothelial cells and their underlying JCT matrix/cells in different flow-type areas in normal human eyes perfusion-fixed at 15 mmHg to understand how changes in cellular connections may play a role in GV and pore formation and regulation of segmental outflow. Our main findings were the following: 1) mean cellular connections were significantly fewer in the cells in high-flow areas compared to those in low- and non-flow areas; 2) GVs of the cells in high-flow areas also had significantly fewer connections beneath them compared to the GVs of the cells in low- and non-flow areas; 3) Type IV GVs had a significantly decreased median number of total connections beneath them per unit volume, compared to Type I GVs; and 4) OL between adjacent IW cells was always 0 µm near B-pores.

This was the first study to quantify the total number of cellular connections on individual SC IW endothelial cells in different flow-type areas. We found that the mean total number of connections on IW cells in high-flow areas was significantly decreased compared to those in low- and non-flow areas (Figure 7A). IW cell-JCT matrix connections and IW cell-JCT cell connections were also significantly decreased in the cells in high-flow compared to those in low- and/or non-flow areas (Figure 7A). These results suggest that the physical separation between the JCT and IW observed in *ex vivo* perfused eyes may be due to decreased cellular connectivity between the IW cells and JCT cells/matrix.

Previous studies have shown that the distribution of flow areas around the circumference of the eye can change under certain conditions. For example, one study used non-diseased human *ex-vivo* donor eyes perfused with fluorescent tracers labeling on day 1 and day 8 of continuous perfusion at 1x or 2x physiologic pressure (Vranka et al., 2020). This study found that over time the amount of these flow areas and their positions could change overtime at 2x physiologic pressure. Previous studies with rho-kinase inhibitors, including Y27632 and netarsudil, found that the amount of high-flow (or active flow) areas around the eye is increased compared to untreated control eyes (Yang et al., 2013; Ren et al., 2016). Based on these observations, low-flow areas do not necessarily remain low-flow areas and could become high-flow areas when induced pharmacologically. Our current study’s findings of fewer connections between IW and JCT in high-flow areas suggest that modulating the cellular connectivity between these two tissues could be a method to increase the amount of EFA around the circumference of the eye.

To better understand GV formation and its relationship to the cellular connections between the IW cells and underlying JCT cells/matrix, we determined the mean number of connections beneath GVs and found that GVs in cells in high-flow areas had significantly fewer connections compared to GVs in cells of low- and non-flow areas (Figure 8B). We also found that the mean percentage of connections under GVs for all IW cells was 39.1% overall compared to 60.9% in areas of the IW cells without GVs. These findings suggest that the IW cells may need to detach from the underlying JCT cells/matrix in areas where GVs will form. Additionally, we determined the ratio of the total number of connections beneath the GVs per unit volume. We used this ratio to account for differences in volumes between the four types of GVs. We found that the ratio of GVs was significantly decreased in Type IV GVs compared to Type I GVs (Figure 8C). Our previous study found that cells perfusion-fixed at 15 mmHg had significantly fewer connections to underlying JCT cells/matrix and significantly larger GVs with I-pores than cells fixed at 0 mmHg, which did not have I-pores (Lai et al., 2019). Another of our previous studies demonstrated that GVs with I-pores have significantly larger volumes and span significantly more sections than those without I-pores and that high-flow areas have significantly more Type IV GVs compared to non-flow areas (Swain et al., 2021). In the context of our previous studies, our current findings may suggest that the number of connections between IW cells and underlying JCT cells/matrix may influence GV size. These findings together support the hypothesis that modulating cellular connectivity may promote the formation of larger Type IV GVs in the IW endothelium and increase AH outflow.

While we did not find a difference in the amount of OL between different flow-type regions, we did find that the OL was always 0 µm on the sections before and after a B-pore appeared. The similarity in OL between flow-type areas was not surprising given that a relatively similar number of B-pores were found in each population of cells from each flow-type area. The finding that OL was always 0 µm before and after a B-pore is consistent with previous studies and the hypothesis that tight junctions simplify and that OL needs to decrease in order to form B-pores in the IW endothelium (Ye et al., 1997; Lai et al., 2019). One previous study in mouse eyes found that targeting tight junctional proteins with small interfering RNA resulted in significantly increased paracellular permeability (Tam et al., 2017). Our findings support the hypothesis that potentially targeting tight junctions to reduce OL may be a strategy for pharmaceutical intervention to promote B-pore formation and increase AH outflow.

We also found that the IW cell dimensions varied among flow-type areas. In high-flow areas, there is lower resistance to AH outflow and a larger pressure difference between anterior chamber IOP and the venous system; thus, more AH flow is directed through these areas. In response to this AH flow and pressure gradient, IW endothelial cells deform and change shape by utilizing a reservoir of excess membrane that is stored in folds, or vesicles, in order to maintain total membrane surface area (Raucher and Sheetz, 1992; Lee and Schmid-Schönbein, 1995; Overby et al., 2009; Pedrigi et al., 2011). Our IW cells from non-flow areas were significantly shorter and narrower in nuclear and non-nuclear areas compared to IW cells in low-flow areas. IW cells from non-flow areas also had smaller volumes compared to IW cells in low- and high-flow areas. These differences may be related to differences in the amount of AH flow across these cells compared to those in high-flow areas. However, with our method, there were various factors that could have influenced the IW cellular dimensions that we were not able to measure, including cytoskeletal dynamics, extracellular matrix stiffness, cell tension, stage of the cell cycle for the IW cells, and fluid shear stress in the lumen of SC, so further investigation is warranted into the factors that impact cellular dimensions. Also, the selected IW endothelial cells in this study were limited to the cells that had their cell bodies completely within the frame of our images and image stacks; therefore, our sample is not fully random. A larger study with more cell and eyes would be needed to confirm this result.

SBF-SEM provided a detailed method to investigate morphological characteristics of individual IW endothelial cells; however, there were some limitations in our study. SBF-SEM is a static imaging modality; therefore, we cannot determine the life cycle of the connections between IW cells and JCT cells/matrix. For example, a projection from an IW cell that is identified as an IW cell-JCT matrix connection may have been connected to a JCT cell process immediately before the eye was fixed. Similarly, for GVs without I-pores, we cannot determine whether a GV simply did not form an I-pore or had formed an I-pore that closed immediately before fixation. Further studies utilizing live cell imaging may further elucidate the dynamic process of I-pore formation. Also, we cannot distinguish the different types of integrins that mediate the different types of IW cell-JCT matrix/cell connections with our SBF-SEM method. Finally, due to the labor-intensive nature of SBF-SEM and manual segmentation of cells, we were only able to capture a relatively small population of 45 cells.

In summary, despite SBF-SEM and manual segmentation being labor-intensive, a major advantage of our technique was that the high-resolution, serial imaging allowed us to investigate the connectivity of the IW with the JCT in 3D and provided volumetric data. In this study, we demonstrated that IW cell-JCT cell/matrix connectivity was significantly decreased in the cells in high-flow areas. The GVs in high-flow areas also had significantly fewer connections beneath them compared to low- and non-flow areas. These results suggest that modulating the IW-JCT and IW-IW connectivity may potentially affect the amount of EFA around the circumference of the eye, and thereby modulate aqueous outflow.

## Data Availability

The original contributions presented in the study are included in the article. Further inquiries can be directed to the corresponding author.

## Abbreviations

AH: aqueous humor; B-pore: intercellular pore; EFA: effective filtration area; GV: giant vacuole; I-pore: intracellular pore; IOP: intraocular pressure; IQR: interquartile range; IW: inner wall; JCT: juxtacanalicular connective tissue; OL: overlap length; POAG: primary open-angle glaucoma; SBF-SEM: serial block-face scanning electron microscopy; SC: Schlemm’s canal; SEM: standard error of the mean; TM: trabecular meshwork.
